# Supplementary material for: Performance of COVID-19 case-based surveillance system in FCT, Nigeria, March 2020 –January 2021
Source: PLoS One. 2022 Apr 14;17(4):e0264839. doi: 10.1371/journal.pone.0264839 (PMC9009682; doi:10.1371/journal.pone.0264839)
Supplement: S1 File — (DOCX) [file pone.0264839.s001.docx]

## FCT COVID-19 Surveillance System Evaluation

## Questionnaire for Stakeholders

**Informed Consent**

I am a Resident of the Nigerian Field Epidemiology and Laboratory Training Program (NFELTP), Cohort 11. This questionnaire is to assess the Knowledge, Attitude and Practice of stakeholders in FCT COVID-19 Surveillance System. Findings will be used to make recommendations for interventions and overall improvement of the system. No names will be taken. All data shall be handled with strict confidence. Please be honest as much as possible in your answers. Thank you

Date: …/…/2021

Initial of respondent certifying that informed consent has been given (State your initials e.g. A.B.C)……………………………………………………………………………………………...

**SECTION A: Socio-demographics**

1. Age: (Age as at last birthday)………………………………………………………
2. Gender……………................................................................................................................
3. Marital Status: Married ( ) Single ( ) Divorced ( ) Widowed ( )
4. Name of Facility/Organization……………………………………………………………
5. Local Government Area of Facility/Organization: ……………………………………….
6. Setting of workplace: Urban ( ) Semi-urban ( ) Rural ( )
7. Type of Facility: Public ( ) Private ( ) NGO ( ) Faith based Organization ( ) Others (specify)…………………………………………………………………………………….
8. Level of Facility: Primary ( ) Secondary ( ) Tertiary ( ) Others ( )
9. Length of experience in organization: (In years) …………………………………………..
10. Designation: Doctor ( ) Nurse ( ) Lab. Scientist ( ) Pharmacist ( ) Radiographer ( ) M&E Officer ( ) Medical Record Officer ( ) Administrative Staff ( ) CHEW ( ) CHO ( ) DSNO ( ) ADSNO ( ) Surveillance Focal Person ( ) SORMAS SSO ( )

Others (specify) ………………………………………………………….

**SECTION B: ATTRIBUTES OF FCT COVID-19 SURVEILLANCE SYSTEM**

**SIMPLICITY**

1. Which kind of forms do you use in data collection? Please tick appropriate form below –

Form A0 (CIF for suspected cases) Yes ( ) No ( )

Form A1 (For confirmed cases-Day 1) Yes ( ) No ( )

Form A2 (For confirmed cases – Day 14-21) Yes ( ) No ( )

Form B1 (For close contacts reporting Day1) Yes ( ) No ( )

Form B2 (For close contacts follow-up Day 14-21) Yes ( ) No ( )

IDSR 002 Yes ( ) No ( )

Not applicable to me Yes ( ) No ( )

Any other (specify)______________________________________

1. Are the forms easy to fill? Yes ( ) No ( )
2. Is the COVID-19 case definition easy to understand (i.e., a case is easily ascertained)

Yes ( ) No ( )

1. Are there COVID-19 Posters displayed in your facility? Yes ( ) No ( )
2. If yes, does it contain the following?
   - - COVID-19 case definition: Yes ( ) No ( )
     - COVID-19 Case Management guidelines: Yes ( ) No ( )
3. Do you report all suspected cases of COVID-19? Yes ( ) No ( )
4. If Yes, how do you report?.....................................................................................
5. Do you detect increase in the number of COVID-19 cases? Yes ( ) No ( )
6. Are there any other organizations apart from State/Federal Ministry of Health/NCDC/WHO involved in receiving your case reports? Yes ( ) No ( )
7. If yes, do they have other forms you have to fill? Yes ( ) No ( )
8. Estimate time spent on collecting data, transferring, entering, editing, storing, analyzing and backing-up data;……………………………………………………………
9. In terms of operation, how easy is it to work within the system i.e., workload, workflow, flow of information and inter unit relationship? Easy ( ) Difficult ( )
10. How many staff are involved in data collection in your facility?…………………
11. Are the number of staff adequate to perform the task? Yes ( ) No ( )
12. If No, how many do you think will be optimal? ………………………….....

**FLEXIBILITY**

1. Do you think that any changes in the COVID-19 Surveillance System can easily be accommodated by the data collection tools? Yes ( ) No ( )
2. Is there a method to report any change or new data element as part of the weekly or monthly report? Yes ( ) No ( )

**DATA QUALITY:**

1. Are there staff dedicated to checking and correcting the completeness and validity of data? Yes ( ) No ( )
2. How is data entered? Manually ( ) Electronically ( ) Both ( )
3. Describe the level of completeness of data generated from the COVID-19 surveillance system: Partially complete ( ) Never complete ( ) Always complete ( )
4. What about the validity of the data generated: Partially accurate ( ) Always accurate ( )
5. Have you been supervised on data management before? Yes ( ) No ( )
6. If yes, how many times in the last 12 months? ...............................................
7. Did you receive written/oral feedback upon completion of the supervision? Yes ( ) No ( )
8. How would you assess the care taken in completing the surveillance forms? Poor ( )

Fair ( ) Good ( ) Very Good ( ) Excellent ( )

**TRAINING**

1. Is training mandatory and a written policy of the system? Yes ( ) No ( )
2. Have you ever been trained on the COVID-19 surveillance? Yes ( ) No ( )
3. If yes, what the type of training?

Informal in-house training ( ) Formal training ( ) Virtual training ( )

1. Has the training improved your performance in COVID-19 surveillance? Yes ( ) No ( )
2. Do you think there is a need for more training? Yes ( ) No ( )
3. If yes, how frequently?....................................................................................

**ACCEPTABILITY**

1. Are you willing to continue to participate in this system? Yes ( ) No ( )
2. Are there any challenges in carrying out your work effectively? Yes ( ) No ( )

If yes, state problems please: …………………………………………….

1. Do you feel the system appreciate you for doing your job? Yes ( ) No ( )
2. What additional support will you need to do your job effectively?……

………………………………………………………………………………

1. Have you ever made suggestions/comments about improving the system? Yes ( ) No ( )
2. If yes, please state these suggestions?...........................................
3. Was your suggestion considered? Yes ( ) No ( )

**TIMELINESS**

1. Are there written policy or agreement on timeliness of data reporting? Yes ( ) No ( )
2. Any there any challenges in sending data on timely basis? Yes ( ) No ( )

If yes, state problems please: …………………………………………….

…………………………………………………………………………..

1. How long does it take to collate data from your department?...........................(In minutes)
2. Do you incur additional cost in doing this? Yes ( ) No ( )
3. If yes, estimate how much on it costs on a monthly basis?.................................................
4. How long does it take to carry out test and issue result? (TAT)………………………….
5. How often do you report? Daily ( ) Weekly ( ) Monthly ( ) Quarterly ( )
6. How fast do you complete your weekly report within the new week? 1st Day ( ) 2nd Day ( ) 3rd Day ( ) 4th Day ( ) 5th Day ( )
7. How soon do you complete your monthly report within the new month?

1st 5 days ( ) End of 1st week ( ) 2nd week ( ) 3rd week ( )

**SENSITIVITY**

1. Is the system able to detect all cases of COVID-19? Yes ( ) No ( )
2. Is the system able to detect new cases? Yes ( ) No ( )
3. Are satisfied with the case definition? Yes ( ) No ( )
4. Are there frequent cases of misdiagnosis? Yes ( ) No ( )
5. Any suggestions for improvement?...................................................................

**REPRESENTATIVENESS:**

1. Do you think the system captures people of all ages? Yes ( ) No ( )
2. Do you think system captures people from all geographical locations? Yes ( ) No ( )

**STABILITY /DATA MANAGEMENT/PLANNED USE OF DATA GENERATED FROM THE SYSTEM:**

1. Do you have dedicated staff for the followings?

Data recording Yes ( ) No ( )

Data storage Yes ( ) No ( )

Data transfers Yes ( ) No ( )

Data analysis Yes ( ) No ( )

Case investigation and sample collection Yes ( ) No ( )

Sample processing Yes ( ) No ( )

Contact tracing Yes ( ) No ( )

POE Yes ( ) No ( )

Case management Yes ( ) No ( )

1. Do you get feedback from the next level? Yes ( ) No ( )
2. If yes, how often? Monthly ( ) Quarterly ( ) yearly ( )
3. How do you protect patient privacy (data confidentiality) ……………………………………………………………………………………………?
4. What do you do with the data generated? ......................................................... ........................................................................................................
5. Has the system in your organization ever been interrupted/nonfunctional due to the following?

Inadequate staff Yes ( ) No ( )

Inadequate fund Yes ( ) No ( )

Stock out of consumables Yes ( ) No ( )

Others (specify)………………………………………………………………………….

1. Do you have stipends to carry out your work? Yes ( ) No ( )
2. Do you think you need more resources? Yes ( ) No ( )
3. If yes, please state the categories of resources and quantity needed ………………………………………………………………………………………………
